# Older adult communication types and emotional well-being outcomes during COVID-19 pandemic

**DOI:** 10.1186/s12877-023-03856-8

**Published:** 2023-03-27

**Authors:** Nicholas Cone, Jeong Eun Lee

**Affiliations:** Human Development and Family Studies, Iowa State University, 2222 Osborn Dr, Ames, IA 50011-1084 United States of America

**Keywords:** COVID-19, Loneliness, Information Communication Technology, Well-being, Social connection

## Abstract

**Background:**

The rationale for the present study is a result of the COVID-19 pandemic, as there are fewer opportunities available for older adults to engage in face-to-face interaction and social activities, which may result in changes in the communication methods with their social contacts. The purpose of this study was to explore the relationship between methods of social connectedness and emotional well-being outcomes among older adults at the start of the pandemic.

**Methods:**

Two thousand five hundred and fifty-eight older adults Medicare beneficiaries (65 + years of age) in the National Health and Aging Trends Study at wave 10 (June 2020 to January 2021) were selected for cross-sectional analysis. Participants were measured on brief questionnaires regarding forms of communication with family and friends before and during the pandemic. Emotional well-being outcomes were measured on single items of sadness/depressed and loneliness; as well as a 6-item Likert scale of anxiety during the pandemic. Paired sample t-tests were utilized to examine the forms of communication between before and during pandemic. Hierarchical regressions were conducted to assess the relationship between forms of communication and emotional well-being outcomes.

**Results:**

We found that there were overall decreases in communication frequency during pandemic. Findings from regression analyses indicated information communication technology (ICT) are associated with negative emotional well-being outcomes, whereas in-person social contact are associated with lower levels of negative affect.

**Conclusion:**

These findings suggest utilizing higher levels of ICT has negative implications for older adults’ emotional well-being, contrasting with the positive implication of in-person contacts. These findings highlight the role of ICT in emotional well-being among older adults during pandemic.

## Background

The Coronavirus Disease 2019 (COVID-19) pandemic has consequences for the health and well-being of aging populations. The pandemic exposes older people to life challenges including disrupted social events, family plans, separation from family and friends, irregular access to necessities, and financial strain because of social distancing guidelines [1, 2].

As a result of physical distancing, older adults are forced to rely on information communication technology (ICT) to interact with their social network members. While multimodal connectedness is dominant in our society, older adults appear to be less multimodal than other age groups due to their lack of technological access and use [3]. Literature has consistently found older adults’ preferred communication methods are in-person communication over ICT (i.e., phone, text message, or video call). Thus, relying on alternative communication methods to substitute in-person contact with others may have negative implications for their emotional well-being [4].

Using the national representative data, National Health Aging Trends Study (NHAT), we examined the association between the level and changes of communication forms and emotional well-being outcomes.

## **Information communication technology and emotional well-being among older adults**

A recent survey by Pew Research Center [5] reported that older adults over the age of 65 are using technology (e.g., internet, home broadband, smartphones, tablets, and social media) less when compared to all adults in the US. Nevertheless, older adults are still showing increases in smartphone and internet usage over the last decades [5]. This increased usage of both internet and smartphone brought both positive and negative impact on the lives of older adults. Digital media has the potential to help older adults overcome social isolation and feelings of loneliness. Scholars argue that the use of ICTs can support older adults in communicating and enables a better quality of life as it allows overcoming social isolation and the feelings of loneliness [6, 7]. That is, higher usage of ICTs among older adults reflects their higher activity in general as well as higher emotional well-being [8, 9]. When older adults are unable to meet their social network members due to illness or transportation issues, they often turn to the internet and skype to enhance their social interaction [10]. Considering this, ICTs have become effective in keeping older adults socially connected. However, these studies were conducted before the pandemic, where older adults had the choice of in-person as well as ICTs to socialize with others.

Despite the new opportunities that ICT may offer to promote active social life [11, 12], there are some factors that would limit the association between ICT and emotional well-being of older adults. First, most studies have not compared the usage of ICT vs. in-person communication methods directly [13], as they often compare users and non-users of ICT when examining the benefits of ICT. Second, there are limited findings addressing the oldest old’s usage of ICT even though young old (65 to 74 years) and oldest old (≥ 85 years) may have different expectations and experiences with ICT. Finally, except for a few studies, study samples were limited to regions, or specific interventions, not necessarily from nationally representative samples [12, 14, 15].

Literature has consistently found older adults’ preferences in terms of communication method. For example, social presence theory [16] posits that ICTs, because of reduced social cues, also reduce the ability to convey and experience interpersonal impression and warmth. That is, when given the choice between ICT and in-person social contact, older adults prefer to socialize in-person [4]. In addition, older adults are likely to use ICT to enhance their in-person contacts or social activities rather than preferred methods of communicating with others [13, 17]. Furthermore, scholars found older adults often have the notion that social relationships using technology are superficial compared to in-person communication and they do not want to rely on online-based communication to form or maintain social relationships [18, 19].

Relatedly, Media Richness Theory [20] posits that communication efficiency between people depends on immediacy of feedback and personal focus. That is, communication that are more person focused (i.e., face-to-face interaction), immediate feedback, and transmitting multiple cues (i.e., facial expression) are considered rich. On the other hand, text-based channels which often do not include personal focus or immediacy of feedback are considered as lean. Therefore, this theory also argues in-person interactions have more richness as personal conversation often convey more personalized information, provides immediate feedback, and utilize multiple cues compared to ICT. As a result, scholars argued the utility of ICT in facilitating meaningful social relationship among individuals, in particular, older adults who are more used to in-person communication [21, 22]. Furthermore, studies have shown that the direct positive association between ICT and well-being could be found for older adults with limited opportunities [6]. Applied to older adults using ICT, for those older adults with ample social resources and in-person opportunities, would not benefit as much as those who could compensate with ICT. These theories of compensatory leveling have empirical findings [23, 24].

Given their preferences in their communication methods, it would be worthwhile to examine the implications of these two different types of communication method on the emotional well-being among older adults; however, studies rarely examined the relative benefits of communication methods for older adults’ well-being.

## Impact of COVID-19 on social interaction among older adults

Given the health risk, Centers for Disease Control and Prevention (CDC) recommended, at the beginning of the outbreak, older adults should stay home and avoid social encounters with others when/if possible [25]. Further, initially nursing homes or assisted living facilities were asked to restrict all visitation from friends and family unless for end of life situations [26]. Frequent social outing spaces such as senior centers and congregate meal sites were closed on a state-to-state basis to reduce the risk of spread based on safety guidelines [25, 27]. Other than public and social outing opportunities, older adults are now forced to stay home for extended time to avoid physical contact with others to avoid the infection risk with the coronavirus. Early national strategies for COVID-19 have ranged from strict control with unlimited resources, relentless contribution with limited resources (i.e., United States - herd immunity), and rough rationality with limited resources [28]. In addition, COVID-19 response strategies dynamically adjust based on new developments [29]. This can be seen as the CDC has recommended vaccine booster shots, and continual mask wearing for vaccinated individuals with the surge of the COVID-19 Delta variant [25].

Since the beginning of the pandemic, the lack of in-person social contact or direct contact with family and social network members results in relying on the usage of ICT. Specifically, to cope with this situation, older adults are now more likely to use video chats (i.e., skype) or text channels (e.g., email or text messaging) instead of in-person meetings. Thus, social/physical distancing due to COVID may have resulted in significant decreases in the level of communication with others regardless of communication methods. That is, assessment of the frequency of communication would reflect older adults’ reduced level of communication and interaction with others.

Furthermore, prior studies have investigated the impact of ICT when it is an option to extend an older adult’s social activities beyond in-person socialization. However, most of the studies focused on how utilizing ICT is beneficial for their emotional well-being without considering relative benefits of in-person communication methods. Moreover, few studies have addressed the impact of limited communication methods as well as changes in the communication methods on the emotional well-being outcomes among older adults.

## The current study

Our study addresses these research gaps and examines the methods of communication older adults use before and during the pandemic. We will also examine the association between the types of communication and emotional well-being outcomes before and during pandemic. Further, we will explore changes in communication methods since the start of the pandemic and its association with emotional well-being.

Based on prior research, we have formulated several following hypotheses.

1. Since the beginning of the pandemic, we expect the total frequency of communication (using both in-person and ICT) with social network members will decrease from before to during the pandemic.
  - It is further expected older adults will report higher levels of ICT communication and lower levels of in-person communication from before to during the pandemic.
2. Older adults using more ICTs will report higher levels of negative emotional well-being outcomes (i.e., depression, anxiety, and loneliness).
  - Those who had more in-person contacts during pandemic will report lower levels of negative emotional well-being outcomes.
3. Changes in the level of communication methods will be associated with emotional well-being outcomes. In particular, higher changes in ICT (i.e., increase in ICT) and those in in-person social contacts (i.e., decrease in in-person social contacts) would be associated with lower emotional well-being.

## Methods

### Participants

The present study utilized data from the National Health and Aging Tends Study (NHATS), which is a nationally representative sample of Medicare beneficiaries aged 65+. The National Health and Aging Trends Study (NHATS) was initiated by the U.S. National Institute on Aging (NIA) in 2008 to guide efforts to reduce disability, maximize health and independent functioning and enhance the quality of life at older ages. NHATS data has three types of downloadable files from https://nhats.org/researcher/data-access: (1) NHATS public data files, (2) National Study of Caregiving and other sensitive files, and (3) restricted files. NHATS conducts annual in-home interviews with over 8000 older adults living in the USA. Initial sampling used a stratified three-stage design: (1) county level, (2) zip code level, and (3) beneficiaries [30]. This sample was refreshed at round 5 (2015) using the same sampling design. The present study utilized round 10 (2020) data to understand how older adults were communicating during the COVID-19 pandemic. Out of 4977 participants who participated in the previous round, 3,257 participants responded to a telephone survey at round 10 (response rate of 82.2%) between June 2020 to January 2021. Of those participants, 2,558 were used based on filtering by the sample person/older adult that completed the survey, compared to a proxy individual helping the older adult. Though many of the variables, or items, in the round 10 data ask about “before” and “during” the COVID-19 pandemic, this is a single cohort of participants that were surveyed at a single timepoint.

The average age was 79.20 years old. Of the participants, 59.1% were female, and 40.9% were male. Regarding marital status, most participants were married (49.1%). Most of the participants identified as White, non-Hispanic (79.4%). Comparing the US older adult population [31] to the present sample suggested slightly more married individuals in our sample. Our sample included 75.1% married men, whereas the older US population was reported at 70%. However, our sample had fewer married women (36.9%) compared to 46% in the older US population. Regarding race/ethnicity, the current sample represents a slightly lower racial and minority populations (19.7%) compared to the older US population (23%). When comparing educational attainment to the national sample, 84.3% of older adults (65+) indicated graduating high school or having more education [32]. Compared to the current sample, combining educational attainment of high school and above high school indicated 90.6%, which is slightly raised to the national sample. The percent of total Medicare enrollment in the United States based on resident population (i.e., states) is 18.4% [33]. Medicare is federal health care coverage primarily for those 65 years and older in the United States (with some exceptions based on disability or condition). Medicare typically consists of three main parts: Part A for hospital insurance (e.g., inpatient, skilled care, and/or hospice), Part B for medical insurance (e.g., outpatient, home health care, medical equipment, etc.), and Part D for drug coverage. Part C is typically provided as bundled Parts A, B, and D through a private company [34]. Table 1 provides more detailed information on the sample.

**Table 1:** Demographic Characteristics

Variable	*N* (2558)	Percent	Range	*M* (*SD*)
Age	2558		69–100	79.20 (6.25)
**Gender**				
Male	1047	40.9		
Female	1511	59.1		
**Education**				
Above high school	823	64.9		
High school	326	25.7		
Below high school	120	9.5		
**Race/Ethnicity**				
White	2030	79.4		
Black	362	14.2		
Other	50	2.0		
Hispanic	87	3.4		
More than one, not Hispanic	2	0.1		
DKRF	27	1.1		
**Marital Status**				
Married	1255	49.1		
Living with Partner	51	2.0		
Separated	26	1.0		
Divorced	332	13.0		
Widowed	816	31.9		
Never Married	78	3.0		

Note. Under race/ethnicity: ‘*Other*’ indicates American Indian/Asian/Native Hawaiian/Pacific Islander/other specify, non-Hispanic and ‘*DKRF*’ indicates no race selected and not Hispanic. Percentages may not add up to one hundred based on rounding error. Frequencies may not add up to total sample based on missing

### Measures

#### Forms of Communication

These are two brief 4-item questionnaires for examining changes in contact with family and friends based on before the COVID-19 outbreak and during the COVID-19 outbreak. The opening question asked, “[Before or During] the COVID-19 outbreak, in a typical week, how often were you in contact with family and friends not living with you?” Participants were asked on four communication categories: ‘*phone calls*,’ ‘*emails/media message*,’ ‘*video calls*,’ ‘*in person visits.*’ Those categories were assessed on a 5-point Likert scale: ‘(1) *at least daily*,’ ‘*a few times a week*,’ ‘*about once a week*,’ ‘*less than once a week*,’ and ‘(5) *never.*’ Values were recoded to indicate less frequency as lower values and higher values as more frequency of that communication form.

We then derived three types of communication scores. First, given the frequency of the communication, using all forms of communication was relevant to our hypothesis; we considered the level of communication frequency scores. Thus, we calculated the total communication frequency by summing all forms of communication before and after the pandemic. Each sum score represents the total communication (regardless of communication methods) that older adults have had with their social network members before and after the pandemic.

Second, to differentiate the four forms of communication categories we further categorized them into ICT and in-person social contact forms. To create an ICT variable, forms of communication of phone calls, media messages, and video calls categories were condensed into a single variable using a mean function. Two types of communication frequency scores of each communication method (i.e., digital and in-person social contacts) were also obtained for before and during pandemic.

Third, to examine the difference in the communication forms (before vs. during pandemic), we calculated the difference scores for these two types of communication methods (in-person vs. ICT). The change in the communication form was created by computing the absolute value between the difference of during and before the COVID-19 outbreak. This variable ranged from zero to four. The higher absolute value in ICT indicates an increase in ICT, whereas a higher absolute value of in-person communication would indicate the decrease in in-person communication.

#### Emotional Well-Being Outcomes

This was assessed on three different outcomes: sadness/depressed, loneliness, and anxiety.

Sadness/depressed was asked through the question, “During the COVID-19 outbreak, in a typical week, how sad or depressed have you felt about the outbreak?” This was assessed on a 4-point Likert scale: ‘(1) *not at all*,’ ‘*mild*,’ ‘*moderate*,’ ‘(4) *severe.*’ Loneliness was asked through, “During the COVID-19 outbreak, in a typical week, how often have you felt lonely?,” which was aligned with the HRS COVID SAQ (Self-Administered leave behind Questionnaire) [35]. This was assessed on a 5-point Likert scale that ranged from ‘(1) *never*,’ ‘*rarely*,’ ‘some days,’ ‘*most days*,’ and ‘(5) *every day*.’

Lastly, anxiety/worry was assessed through a 6-item scale, which was adapted from the PTSD-8 scale [35, 36], through the question, “During the COVID-19 outbreak, how much of the time have the following symptoms bothered you?” Participants were asked on, “Recurring thoughts about the outbreak and its effects,” “Recurring nightmares about the outbreak and its effects,” “Avoiding activities that remind you of the outbreak and its effects,” “Avoiding thoughts or feelings about the outbreak and its effects,” “Feeling jumpy or easily startled,” and “Feeling on guard.” This was assessed on a 4-point Likert scale that ranged from ‘(1) *not at all*,’ ‘*rarely*,’ ‘*sometimes*,’ ‘(4) *most of the time.*’ In the present study, anxiety was assessed for factorability of the 6-item scale, using principal axis factoring and ProMax rotation, which a single solution was found. Factor loadings indicated acceptable weights. As this was an adapted PTSD-8 scale for COVID-19 specific data, this is a preliminary factory analysis. All values for these outcomes were recoded to indicate less frequency as lower values and higher values as more frequency of that communication form. The Cronbach’s alpha in the present study is 0.83. Researchers indicated similar Cronbach’s alphas at 0.83, 0.84, 0.85 in three different samples [36].

#### Demographics

Gender was coded as male (1) and female (2). Marital status was assessed on 6 categories (e.g., married, living with a partner, separated, divorced, widowed, and never married). This item was coded as either married/together (1) or not married (0). Age of participants was collected as a continuous numerical variable. Education was assessed on 9 categories: no schooling completed, 1st-8th grade, 9th-12th grade, high school (GED), vocational, some college, associate degree, bachelor’s degree, and master’s/PhD degree. Education was recoded to three groups: (0) below high school, (1) high school, (2) above high school. Race/ethnicity were assessed on 6 categories (e.g., White, Black, Other – American Indian/Asian/Native/Hawaiian/Pacific Islander, Hispanic, more than one, and DKRF – No race indicated). For analysis, race/ethnicity was dummy coded for White, Black, and Hispanic.

### Data Analysis

All data transformations and data analyses were conducted using IBM SPSS package, version 26 [37]. Descriptive statistics were performed by computing frequency analyses or means and standard deviations. To examine the first hypothesis, a paired sample t-test was utilized to examine the forms of communication between pandemic measurement times (i.e., before and during). To examine the second and the third hypotheses, three hierarchical regressions were conducted to assess the relationship between forms of communication (e.g., ICT and in-person social contact) and emotional well-being outcomes (e.g., loneliness, sadness/depressed, and anxiety). Forms of communication during the outbreak were utilized as independent variables, whereas the emotional well-being outcomes were dependent variables. These regressions were structured in blocks: (1) covariates, (2) forms of communication, and (3) changes in the forms of communication. We included covariates that might shape these experiences, including gender, age, race, marital status, and education. In the current data, the outcome variables skewness ranged from − 0.54 to 0.68, and the kurtosis ranged from − 0.20 to − 0.01, which are within acceptable range [38]. Model 3 is not shown in the final tables as we trimmed the model where the block was not significant (However, this is available on request). Missing data was handled through SPSS listwise deletion.

## Results

The first hypothesis considered the level of communication among older adults. Consistent with our first hypothesis, paired t-test analyses found significant differences in the use of all forms of communication between COVID measurement times. Results indicated a difference, *t*(2438) = 14.27 *p* < .001, between before COVID (*M* = 3.11) and during COVID (*M* = 2.96). This suggests participants experienced an overall decrease in their communications to family and friends from before to during COVID. In addition, hypothesis 1a was partially supported through a significant difference in ICT methods, *t*(2410) = 3.30, *p* < .001), and in-person communication, *t*(2332) = 23.84, *p* < .001 (see Table 2). This suggests that when differentiating communication methods separately, older adults reported decreases between before to during the COVID outbreak for ICT and in-person communication methods.

**Table 2:** Forms of Communication Mean Difference

Variable	N	Range	*M* (*SD*)	*t*
**ICT**				
Before COVID	2514	1–5	3.15 (0.92)	3.30***
During COVID	2438	1–5	3.11 (0.95)	
**In-person social contact**				
Before COVID	2480	1–5	3.00 (1.16)	23.84***
During COVID	2359	1–5	2.49 (1.14)	
**Change (During – Before)**				
ICT	2411	0–4	0.32 (0.47)	
In-person social contact	2333	0–4	0.72 (0.93)	
**Forms of communication (Mean)**				
Before COVID	2533	1–5	3.11 (0.82)	14.27***
During COVID	2457	1–5	2.96 (0.82)	

*Note.* Measurement scale is a 5-point Likert: ‘(1) *never*,’ ‘*less than once a week*,’ ‘*about once a week*,’ ‘*a few times a week*,’ and ‘(5) *at least daily.*’

ICT = Information Communication Technology

*p < .05. **p < .01. ***p < .001.

Our second hypothesis examined the relationship between communication forms and emotional well-being outcomes. Findings indicated support on ICT to anxiety, and sadness/depressed mood. Moreover, there was support for in-person social contact on loneliness and anxiety.

In terms of depressed mood, our hypothesis was partially supported. There was a positive association between ICT and sadness/depressed mood during the outbreak (β = 0.07, *p* < .05, see Table 3). However, we found no significant association between in-person communication and depressed mood (coefficient report, see Table 3). This finding supports the hypothesis that older adults utilizing more ICT would indicate higher negative emotional well-being outcomes; however, the level of in-person social contact and depressive symptoms were not significantly associated.

Regarding anxiety, results were consistent with our second hypothesis. there was a positive relationship between using ICT, (β = 0.12, *p* < .001, see Table 3). This suggests older adults reported using ICT during the outbreak reported higher levels of anxiety. Moreover, there was a negative relationship between in-person social contact and anxiety, (β = -0.08, *p* < .001, see Table 3). The findings indicate support for increases in negative emotional well-being outcomes with increased ICT use, and the reduction of these outcomes with the use of in-person social contact.

**Table 3:** Forms of Communication Predicting Sad/Depressed Feelings, Anxiety

	Sadness/Depressed			Anxiety		
Variable	*B*	*SE B*	β	*B*	*SE B*	β
Age	0.01	0.00	0.07*	0.00	0.00	0.03
Gender	0.29	0.05	0.18***	0.19	0.04	0.15***
Marital Status	-0.01	0.05	0.00	0.02	0.04	0.01
Education	0.08	0.04	0.07*	0.02	0.03	0.02
White, non-Hispanic	-0.17	0.13	-0.09	-0.20	0.11	-0.13
Black, non-Hispanic	-0.21	0.14	-0.10	-0.04	0.12	-0.02
Hispanic	0.09	0.17	0.02	0.10	0.15	0.03
During Pandemic ICT	0.05	0.03	0.07*	0.08	0.02	0.12***
During Pandemic In-person social contact	-0.03	0.02	-0.04	-0.04	0.02	-0.08*
ICT Change						
In-person Change						
R^2^		0.053			0.058***	

*Note.* Age is defined as continuous. Gender is defined as male (1) and female (2). Marital status is defined as single (0) and married/together (1). Education is defined as (0) below high school, (1) high school, and (2) above high school. Race/Ethnicity is dummy coded (0/1)

ICT = Information Communication Technology

*p < .05. **p < .01. ***p < .001.

Regarding loneliness, there was partial support for our second hypothesis regarding in-person social contact. In-person social contact, (β = − 0.08, *p* < .001, see Table 4) was negatively associated with loneliness. This finding indicates individuals using in-person social contact reported lower levels of loneliness. We did not find a significant association between ICT and loneliness.

**Table 4:** Forms of Communication predicting loneliness

	Model 1			Model 2			Model 3		
Variable	*B*	*SE B*	β	*B*	*SE B*	β	*B*	*SE B*	β
Age	0.00	0.01	0.01	0.00	0.01	0.01	0.00	0.01	0.01
Gender	0.30	0.06	0.15***	0.29	0.06	0.15***	0.29	0.06	0.15***
Marital Status	-0.33	0.06	-0.17***	-0.35	0.06	-0.18***	-0.35	0.06	-0.18***
Education	-0.02	0.05	-0.01	-0.04	0.05	-0.02	-0.04	0.05	-0.02
White, non-Hispanic	0.12	0.17	0.05	0.14	0.17	0.06	0.17	0.17	0.07
Black, non-Hispanic	-0.16	0.18	-0.06	-0.16	0.18	-0.06	-0.13	0.18	-0.05
Hispanic	0.05	0.22	0.01	0.04	0.22	0.01	0.04	0.22	0.01
During Pandemic ICT				0.03	0.03	0.03	0.04	0.03	0.03
During Pandemic In-person social contact				-0.07	0.03	-0.08	-0.06	0.03	-0.08*
ICT Change							0.15	0.07	0.06*
In-person Change							-0.01	0.03	-0.01
R^2^		0.072***			0.079*			0.082	

*Note.* Age is defined as continuous. Gender is defined as male (1) and female (2). Marital status is defined as single (0) and married/together (1). Education is defined as (0) below high school, (1) high school, and (2) above high school. Race/Ethnicity is dummy coded (0/1)

ICT = Information Communication Technology

*p < .05. **p < .01. ***p < .001

Regarding our third hypothesis, there was support for loneliness outcome on ICT change. Results indicated positive association between ICT change (β = 0.06, *p* < .05, see Table 4) and loneliness. This indicates that older adults who report more changes (i.e., increases) in ICT report higher loneliness. However, there was no support for changes in communication for ICT or in-person social contact for emotional well-being outcomes of anxiety, or sad/depressed mood.

## Discussion

Unprecedented challenges of the COVID-19 global pandemic have a profound impact on older adults’ health and well-being with social distancing guidelines. The purpose of this study was to examine the association between forms of communication on emotional well-being outcomes (e.g., loneliness, sad/depressed, and anxious feelings) between time points of before and during the pandemic. Our findings on the level of communication and emotional well-being shed light on the literature by highlighting the association between older adults’ social connection amid the pandemic and their emotional well-being. In addition, the role of ICT emerged as challenges as well as opportunities during pandemic.

Our findings indicated that overall levels of communication with family and social network members have decreased during the pandemic when compared with before the pandemic. This change is bound to have emotional ramifications for older adults who value social connection with family and friends in late life [39]. Our finding suggests that fewer frequent communication during the pandemic, especially decreases in-person social contact with their social network members contributes to increases in negative emotional well-being outcomes. Furthermore, consistent with prior literature on the quantifying social network, our findings show that this decrease has implication on their mental health [40, 41]. Taken together, communication forms take on a complex role in the lives of older adults during the pandemic.

Though older adults indicated using ICT on average at least once a week, they may have found themselves forced to utilize ICT. As the social presence and media richness theories [16, 20] argued, older adults who prefer rich cues and context in their communication may find it difficult to maintain their interpersonal relationships.

Our findings indicate that the higher levels of ICT during a pandemic have negative implications for emotional well-being such as sadness/depressed, or anxious outcomes. This finding is inconsistent with prior findings on the positive benefits of using ICT on their mental health before pandemic [3, 6]. There are plausible reasons for how using more ICT during the pandemic is associated with emotional well-being during pandemic. On one hand, using ICT could alleviate social isolation among older adults [13] by providing social connection with the outside world, gaining social support, and engaging in social activities while maintaining physical distance. On the other hand, this higher level of using ICT could reflect their limited choices in communicating with their social contacts. Furthermore, combining with reduced in-person social contact, using ICT may have reminded older adults of pandemic and associated consequences because ICT acts as ways to relay positive or negative news of the pandemic. Older adults may feel connected on a basic level to family and friends, but quality of information and cues using ICT may be lacking personal warmth and connection as media richness theory and social presence theory posits [16, 20]. Finally, prior studies supporting the prosocial effect of ICT often compared using ICT versus not using ICT, leading to positive outcomes. Given that in-person social contacts were associated with lower levels of anxiety, our findings support the notion that older adults are more likely to experience distress due to relying on ICT in lieu of in person contacts.

Compared to emotional well-being (i.e., depressed/anxious and sadness) outcomes, we found that higher ICT was not associated with loneliness. Although loneliness and depressed mood are often associated among older adults, they are not necessarily the same; a person can feel lonely even when they are not depressed. Rather, our findings show that not the level of ICT, but change in the ICT contributes to the feelings of loneliness. Combined with the result showing that lower levels of in-person social contacts were associated with loneliness, we argue that it is difficult to maintain quality relationships though ICT. This further suggests older adults could have utilized their preferred communication method (i.e., in-person) to amplify their in-person contacts prior to the pandemic, which becomes unavailable during pandemic.

Taken together, though older adults may feel ICT lacks in relationship quality, the sheer nature of being able to communicate with family and friends may provide some level of relief. However, it is important to recognize the differential associations when using ICT. Though older adults might not prefer ICT, as seen through our results, it becomes clear that it is a necessary lifeline during the pandemic.

Limitations and future directions for the present study include several considerations. First, emotional well-being may not be the direct result of communication methods. It is possible COVID-19 specific stressors (e.g., health challenges, personal loss, or lack of physical activities) may contribute to lower emotional well-being outcomes. Furthermore, many emotional well-being items were limited to a single item question, which limits the ability to capture a construct. Second, the current data did not assess technology acceptance levels, or comfort of using technology, which could confound the relationship between communication forms and emotional well-being. In addition, the current dataset does not provide items on technology literacy, which could influence our findings as some older adults are more, or less, familiar with ICTs. Third, all forms of technological communication (e.g., phone, video call, and email) were combined into one category. Although this is typically standard, they may have differential implications for their mental health [7, 12, 13]. Fourth, the relationship quality between participants and their social network members such as family and friends were not assessed. As prior studies suggested, the quality of the relationship may matter more than the frequent communication or social exchanges with social network members. Fifth, there was a sample selection bias of only older adults that completed the survey themselves. Relatedly, the participants sampled in the present data are only Medicare beneficiaries, which may limit the ability to draw conclusions to all older adults. Lastly, the study is semi cross-sectional. Though it utilizes a before and during time wording, this data was retrieved from a single round. Future studies should attempt to use longitudinal data to examine multi-itemed emotional well-being variables (UCLA Loneliness Scale, Patient Health Questionnaire-9, or Generalized Anxiety Disorder Assessment-7), and potential other stressors (i.e., social isolation), as well as controlling for variables such as relationship quality of targeted communication groups and technology acceptance levels.

## Conclusion

In the past several decades, older adults are increasing in technology adoption rapidly; however, some still prefer face-to-face communication. Given the lack of choices, older adults may have felt better with having alternative options, but overall communication with others has been reduced and this limited choice to communicate with others may have affected their mental health. Our study highlights the distinctive role of ICT and in-person contacts on emotional well-being among older adults during pandemic.

## Data Availability

The dataset supporting the conclusions of this article are available at https://nhats.org/researcher/data-access.
